# Remission rate of type 2 diabetes mellitus following multidisciplinary management in the diabetes reversal clinic and its predictive factors: a real-world study

**DOI:** 10.3389/fendo.2026.1781655

**Published:** 2026-04-14

**Authors:** Ruilin Shen, Zihan Zhang, Hongping Sun, Doudou Li, Qing Cheng, Jianyin Zhou, Dengyue Zhu, Shuhang Xu, Guofang Chen, Liu Chao

**Affiliations:** 1 Department of Endocrinology, Affiliated Hospital of Integrated Traditional Chinese and Western Medicine, Nanjing University of Chinese Medicine, Nanjing, China; 2 Nursing Department, Affiliated Hospital of Integrated Traditional Chinese and Western Medicine, Nanjing University of Chinese Medicine, Nanjing, China; 3 Department of Nutrition, Affiliated Hospital of Integrated Traditional Chinese and Western Medicine, Nanjing University of Chinese Medicine, Nanjing, China; 4 Department of pharmacy, Affiliated Hospital of Integrated Traditional Chinese and Western Medicine, Nanjing University of Chinese Medicine, Nanjing, China

**Keywords:** lifestyle intervention, multidisciplinary management, predictive factors, remission rate, type 2 diabetes mellitus

## Abstract

**Objective:**

To evaluate the effects of multidisciplinary management in the Diabetes Reversal Clinic on the remission of type 2 diabetes mellitus (T2DM), and explore the predictive factors for remission of diabetes.

**Methods:**

This was a real-world, single-arm observational study. Patients with T2DM who received remission-oriented treatment at the Diabetes Reversal Clinic and followed up regularly for more than 24 weeks were included. The primary outcome was the remission rate of T2DM, and the secondary outcomes were changes in fasting blood glucose (FBG), 2-hour postprandial blood glucose, HbA1c, blood lipids, and body composition. Differences in clinical characteristics between the remission and non-remission groups were analyzed. The multivariate logistic regression analysis was performed to screen predictive factors.

**Results:**

The remission rate was 29.24% (50/171) after multidisciplinary management in the Diabetes Reversal Clinic. The remission group was younger, had a shorter duration of diabetes, used fewer types of medications at baseline, had a lower FBG and HbA1c, a higher β-cell function index HOMA-β, and a lower triglyceride level. The multivariate logistic regression analysis revealed that age (OR = 0.93, 95% CI: 0.89-0.97, *P* = 0.002), type of medications at baseline (OR = 0.45, 95% CI: 0.26-0.77, *P* = 0.004), baseline FBG (OR = 0.64, 95% CI: 0.46-0.89, *P* = 0.008), and weight loss magnitude (OR = 1.15, 95% CI: 1.01-1.31, *P* = 0.038) were independent predictors of T2DM remission.

**Conclusion:**

In this real-world study, the remission rate of T2DM patients who visited the Diabetes Reversal Clinic for more than 24 weeks was 29.24% (50/171). Younger age, shorter disease duration, fewer baseline medications, lower FBG and HbA1c, higher HOMA-β, lower triglycerides, and greater weight loss were associated with remission of T2DM. Among them, age, baseline medication type, baseline FBG, and weight loss were identified as factors independently associated with remission. However, due to the single-arm observational design, causality cannot be established, and further prospective controlled trials are required to confirm these findings.

## Introduction

In the management of type 2 diabetes mellitus (T2DM), remission is a goal which can delay the deterioration of diabetic complications, prolong lifespan, and reduce long-term medical costs (1, 2). Remission is associated with a lower mortality and fewer microvascular complications, including diabetic retinopathy, nephropathy and neuropathy (3–6). Moreover, for patients presenting remission, the discontinuation of hypoglycemic medications prevents series adverse reactions, like hypoglycemia, and enhances patients’ compliance. Besides, anxiety and depression can be relieved as diabetes regresses, thus favoring patients’ mental health and quality of life (7).

Conventional management often fails to achieve the goal of diabetes remission. The remission rate is usually less than 5% in T2DM patients managed by routine lifestyle interventions and medications (8). Intensive interventions are more effective to realize the remission of T2DM. Evidence from the DiRECT study shows a high remission rate of 46% in T2DM patients diagnosed within the 6 years after a 12-month low-calorie diet intervention and weight management (9). The DIADEM-I study demonstrated that intensive lifestyle interventions for one year bring remission in 61% of young early-stage diabetes patients from the Middle East (10). Weng et al. validated the excellent performance of intensive insulin therapy combined with lifestyle interventions in achieving a 1-year remission rate of higher than 30% (11, 12). Metabolic surgery has also proven effective to induce diabetes remission. Jia et al. reported complete and partial remission rates of 54.4% and 50.0%, respectively, in obese people with T2DM after 5 years of gastric bypass and sleeve gastrectomy (13).

While offering strengths in diabetes remission, these intensive interventions are challenged by many factors in their wider uses. First, a strict restriction of calories in diets can hinder the long-term adherence (14). Second, the intensive insulin pump is expensive, technically demanded, and associated with an increased risk of hypoglycemia. Third, surgery as an invasive procedure can only be performed in diabetic patients with strict indications, often followed by postoperative complications and long-term adverse events (15). Currently, the benefits of intensive interventions on diabetes remission been predominantly described in rigorously designed controlled clinical trials. How to increase the diabetes remission rate in the real world is worth exploring.

While multidisciplinary team (MDT) management models may be associated with increased T2DM remission rates in outpatient settings, relevant clinical evidence remains scarce and lacks sufficient validation in real-world cohorts. Recently, a Canadian study reported that a multidisciplinary lifestyle intervention (predominantly Mediterranean diet-based nutritional counseling and personalized exercise) was associated with a 100% (11/11) remission rate in drug-naive T2DM patients and a 2% (1/43) remission rate in drug treated T2DM patients (16). Another retrospective evaluation showed that 1-year online multidisciplinary lifestyle intervention resulted in 24% remission in nonobese Indian T2DM patients (17). However, there is no relevant report on whether it is feasible and effective to improve the remission rate of T2DM by implementing multidisciplinary management in outpatient services in Chinese tertiary hospitals.

Predictive factors for diabetes remission can be used to recognize individuals who will experience diabetes remission, guide interventional regimens, and implement precision treatment. Previous evidence shows that diabetes remission via intensive lifestyle interventions is closely linked with weight loss, baseline glycated hemoglobin (HbA1c) and the duration of diabetes (3). During the insulin intensive therapy, a lower baseline HbA1c, a lower average blood glucose level, a better recovery of first-phase insulin secretion capacity, a lower demand for exogenous insulin, and a shorter disease duration all predict a greater likelihood of remission (18, 19). A low baseline fasting blood glucose (FBG) and a short duration of diabetes predict a high diabetes remission rate after metabolic surgery (13). Most of the above predictive factors are derived from intensive interventions in strictly designed clinical trials. It remains urgent to explore more universal and practical models that can be used for predicting diabetes remission in the real world.

In the present study, we investigated the impact of a multidisciplinary management model in the Diabetes Reversal Clinic on T2DM remission rate, and explored independent factors that can predict remission. We hypothesized that this integrated, sustainable, multidisciplinary model would achieve a clinically meaningful remission rate, and that baseline and modifiable clinical factors might be independently associated with remission. Our findings may support the wider replication of this practical model for T2DM reversal in routine clinical practice.

## Methods

### Study design and participants

This was a single-arm, observational study. A total of 171 T2DM patients were enrolled, with an age of 18–75 years, a BMI of 18.5-40.0 kg/m^2^, and an HbA1c<14%, who visited the Diabetes Reversal Clinic from April 12, 2022 to December 31, 2024. Only patients with a minimum of 24 weeks of complete follow-up data were included in the final statistical analysis to ensure the integrity of intervention implementation and outcome assessment. Excluded were those with acute or severe chronic diabetes complications, a history of pancreatitis, medullary thyroid carcinoma, recurrent genital candidiasis, fractures, and malignancies, severe organ dysfunction, severe cognitive dysfunction, language disorder, and mental illnesses. Pregnant or breastfeeding women were also excluded. This study was approved by the Ethics Committee of the Affiliated Hospital of Integrated Traditional Chineses and Western Medicine (No. 2021-LWKYZ-002).

### Multidisciplinary management in the Diabetes Reversal Clinic

An MDT was set up, consisting of endocrinologist, cardiologist, nutritionist, rehabilitation specialist, clinical pharmacist and nurse, with clearly defined and standardized professional roles for each member to ensure whole-process collaborative management. Endocrinologist led the MDT, conducted comprehensive baseline assessments, formulated individualized treatment plans (lifestyle and/or medication strategies), and monitored therapeutic response and safety events. Cardiologist assessed and managed cardiovascular comorbidities (e.g., hypertension, dyslipidemia) to optimize cardiometabolic risk control and integrate cardiovascular care into diabetes management. Clinical nutritionist designed personalized dietary intervention plans, provided one-on-one dietary counseling and education, and monitored dietary adherence. Rehabilitation specialist performed baseline physical fitness evaluations, prescribed individualized exercise regimens based on patient tolerance and comorbidities, guided safe exercise implementation and progression. Clinical pharmacist guided the safety and rational use of hypoglycemic medication, and hypoglycemia recognition and management. Diabetes specialist nurse coordinated regular follow-up visits, guided self-monitoring of blood glucose, and delivered diabetes self-management education.

Dietary intervention recommended to follow a caloric restriction diet, with a target deficit of 500–750 kcal/d from daily calorie intake based on age, sex, BMI, and physical activity level. Encourage high-quality protein intake (e.g., 1–2 egg for breakfast, 100–160 g of lean meat or fish/shrimp for lunch and dinner) and fiber-rich foods (≥300–500 g of vegetables per day with half dark greens). Refined carbohydrates, added sugars, and saturated/trans fats were strictly limited, with daily vegetable oil intake controlled to 25–30 ml and salt intake <5 g. Between-meal snacks was discouraged. All patients were prescribed a combined regimen of aerobic and resistance training, with individual adjustments based on baseline physical fitness, comorbidities, and exercise tolerance. Moderate-intensity aerobic exercise: at least 150 minutes per week, divided into ≥3 non-consecutive sessions, with modalities including brisk walking, cycling, and swimming. Resistance training: 2–3 sessions per week (≥48 hours of rest for the same muscle group), with modalities including bodyweight exercises (squats, planks, push-ups) and elastic band training, targeting major muscle groups with 3 sets of 10–15 repetitions per movement. Adherence was monitored through self-reported logs and periodic counseling sessions.

Medication titration and withdrawal followed a predefined stepwise protocol. Once patients achieved the glycemic targets (FBG 3.9-5.6 mmol/L and 2-hour PPG 5.0-7.8 mmol/L) for at least two consecutive visits, medication doses were gradually reduced at 4–6 week intervals. For patients on combination therapy, medications were withdrawn sequentially rather than simultaneously. The sequence of reduction prioritized agents with higher hypoglycemic risk (e.g., insulin, sulfonylureas) first. Drug withdrawal was considered when HbA1c remained below 6.0% on the minimal effective dose for at least 4 weeks.

During the follow-up, one or two times of visits were required within the first three months, and once every one to three months later. HbA1c were measured every three months. For individuals with drug withdrawal, the blood glucose level was reexamined with an interval of three months, and then six months until the disease stabilized. Recurrence of hyperglycemia was considered as a condition in which an FBG persistently remained above 7.0 mmol/L or 2-hour PPG remained above 10 mmol/L for one week and longer, and treated following the *2020 Guideline for the Prevention and Treatment of Type 2 Diabetes Mellitus in China*.

### Outcomes

T2DM remission rate, as the ratio of patients showing remission to the total participants, was the primary outcome. Diabetes remission referred to a state of HbA1c <6.5% maintained for a minimum of three months without the use of hypoglycemic drugs, consistent with the 2021 ADA consensus report definition (20). Of note, the consensus group intentionally avoids distinguishing between partial and complete remission, citing concerns that such distinctions could introduce ambiguity in policy and clinical coding. Therefore, our definition aligns with the single consensus criterion without subclassification.

Indicators of glucose metabolism, blood lipids, and body composition were secondary outcomes. Glucose metabolism indicators consisted of FBG, 2-hour PPG, HbA1c, fasting insulin (FINS), fasting C-peptide (FC-P), homeostasis model assessment of insulin resistance (HOMA-IR), and homeostasis model assessment of β-cell function (HOMA-β). Indicators of blood lipids included total cholesterol (TC), triglyceride (TG), high-density lipoprotein cholesterol (HDL-C), and low-density lipoprotein cholesterol (LDL-C). Body composition indicators compromised body weight (BW), waist circumference (WC), BMI, waist-to-height ratio (WHtR), waist hip rate (WHR), body fat mass, body fat percentage, skeletal muscle content, and visceral fat area (VFA).

### Statistical analysis

SPSS 26.0 was used for data processing. Measurement data within a normal distribution were expressed as mean ± standard deviation (SD), and compared using the t-test. Those out of a normal distribution were expressed using median (P25, P75), and compared via the rank sum test. Enumeration data between groups were compared by the Chi-square test or Fisher’s exact test. Multivariate logistic regression analysis was used to identify independent factors associated with T2DM remission. Independent variables were selected through a two-step process: (1) variables with a statistical trend (*P* < 0.1) in univariate logistic regression analysis were included as candidates; (2) additional clinically relevant factors identified from previous literature were also incorporated (e.g., duration of disease). Prior to model construction, multicollinearity among candidate variables was assessed using the variance inflation factor (VIF), with variables exhibiting multicollinearity (VIF≥2) excluded. The performance of the final multivariate logistic regression model was evaluated using: (1) the area under the receiver operating characteristic curve (AUC) to assess discriminatory ability; (2) the Hosmer-Lemeshow goodness-of-fit test to evaluate model calibration; and (3) Bootstrap resampling (1000 replicates) for internal validation to assess model stability and potential overfitting. *P* < 0.05 suggested a significant difference.

## Results

### Diabetes remission rate in Diabetes Reversal Clinic

The cohort of 171 T2DM patients who regularly visited the Diabetes Reversal Clinic for 24 weeks consisted of 68 (39.77%) female and 103 (60.23%) male patients, with an average age of 50.34 ± 11.98 years and a median disease duration of 3.00 (1.00, 7.00) years. Nearly half of them (69/171, 40.35%) experienced comorbidities, with hypertension, hyperlipidemia and thyroid diseases being the most common. An average of 1.54 ± 1.04 types of antidiabetic drugs were medicated at baseline.

Fifty patients finally presented remission, with an overall remission rate of 29.24%. Categorized by medications, basal insulin, GLP-1RA, basal insulin+GLP-1RA and oral hypoglycemic drug groups consisted of 41 (23.98%), 34 (19.88%), 11 (6.43%) and 85 (49.71%) patients, respectively. The highest diabetes remission rate was seen in the hypoglycemic drug group (37.65% [32/85]), followed by GLP-1RA group (35.29% [10/34]), basal insulin group (17.07% [7/41]), and basal insulin+GLP-1RA group (9.09% [1/11]) (**Table 1**).

**Table 1:** Therapeutic regime and remission rate of 171 T2DM patients.

Therapeutic regime	All patients	Patients in remission
Basal insulin (n, %)	41 (23.98)	7 (17.07)
GLP-1RA (n, %)	34 (19.88)	10 (35.29)
Basal insulin+GLP-1RA (n, %)	11 (6.43)	1 (9.09)
Oral hypoglycemic drugs (n, %)	85 (49.71)	32 (37.65)

T2DM, type 2 diabetes mellitus; GLP-1RA, glucagon-like peptide-1 receptor agonist.

### Clinical characteristics of T2DM patients in remission and non-remission groups

After 24 weeks of regular multidisciplinary management in the Diabetes Reversal Clinic, the patients with diabetes were assigned into the remission group (n=50) and non-remission group (n=121). Compared with those in the non-remission group, T2DM patients in the remission group were significantly younger, with a shorter duration, fewer types of hypoglycemic drugs, lower baseline FBG, HbA1c and TG, and a higher baseline HOMA-β (*P* < 0.05, **Table 2**).

**Table 2:** Baseline characteristics of T2DM patients in the non-remission group versus the remission group.

Parameters	Non-remission (n=121)	Remission (n=50)	*P*
Demographic data			
Gender (n, %)			0.518
Male	71 (58.68)	32 (64.00)	
Female	50 (41.32)	18 (36.00)	
Age (years)	52.24 ± 11.27	45.74 ± 12.52	0.001
Duration of disease (years)	4.00 (2.00, 8.00)	1.00 (0.23, 3.00)	0.000
Comorbidities (n)	1.00 (0.00, 1.50)	0.50 (0.00, 1.00)	0.133
Hypoglycemic drugs (n)	2.00 (1.00, 2.00)	1.00 (0.00, 1.25)	0.000
Vital signs			
SBP (mmHg)	129.15 ± 16.62	127.12 ± 11.25	0.357
DBP (mmHg)	77.10 ± 10.57	76.86 ± 10.71	0.893
HR (bpm)	81.76 ± 11.27	81.67 ± 12.02	0.946
Glucose metabolism			
FBG (mmol/L)	8.58 ± 2.53	7.11 ± 1.98	0.000
HbA1c (%)	7.95 ± 1.59	7.40 ± 2.07	0.001
FINS (μU/mL)	9.25 ± 5.06	11.54 ± 7.69	0.293
HOMA-β	38.09 (23.83, 54.72)	54.18 (39.32, 90.25)	0.002
HOMA-IR	3.14 (2.19, 4.17)	3.23 (1.91, 5.41)	0.920
FC-P (pmol/L)	744.13 ± 312.86	842.11 ± 424.03	0.274
Blood lipids			
TC (mmol/L)	4.78 ± 1.08	4.67 ± 1.25	0.570
TG (mmol/L)	1.54 (1.09, 2.39)	1.08 (0.68, 2.08)	0.010
HDL-C (mmol/L)	1.16 (1.00, 1.39)	1.07 (0.95, 1.31)	0.201
LDL-C (mmol/L)	2.97 ± 0.83	2.94 ± 0.96	0.827
Body composition			
BW (kg)	69.13 ± 11.65	71.81 ± 14.14	0.201
WC (cm)	89.01 ± 8.74	85.40 ± 13.28	0.093
BMI (kg/m^2^)	24.63 ± 3.05	25.17 ± 3.76	0.596
WHtR	0.53 ± 0.05	0.49 ± 0.05	0.072
Body fat mass (kg)	18.40 ± 5.08	20.61 ± 8.83	0.435
Body fat percentage (%)	27.67 ± 6.89	28.01 ± 9.01	0.857
Skeletal muscle content (kg)	26.52 ± 5.61	28.88 ± 6.23	0.068
WHR	0.88 ± 0.04	0.89 ± 0.05	0.428
VFA (cm^2^)	97.27 ± 41.21	96.74 ± 34.66	0.684

T2DM, type 2 diabetes mellitus; SBP, systolic blood pressure; DBP, diastolic blood pressure; HR, heart rate; bpm, beats per minute; FBG, fasting blood glucose; HbA1c, glycated hemoglobin; FINS, fasting insulin; HOMA-β, homeostasis model assessment-beta cell function; HOMA-IR, homeostasis model assessment-insulin resistance; FC-P, fasting C-peptide; TC, total cholesterol; TG, triglyceride; HDL-C, high-density lipoprotein cholesterol; LDL-C, low-density lipoprotein cholesterol; BW, body weight; WC, waist circumference; BMI, body mass index; WHtR, waist-to-height ratio; WHR, waist hip rate; VFA, visceral fat area.

Indicators of glucose metabolism, blood lipids, and body composition were compared before and after 24 weeks of multidisciplinary management between groups (**Tables 3**, **4**). Post-treatment WC, WHtR, FBG, HbA1c and HOMA-IR were significantly lower, while HDL-C was significantly higher than those at baseline in both groups (*P* < 0.05). In addition, a significant elevation of HOMA-β and a significant reduction of TG were seen in the non-remission group (*P* < 0.05). BW and body fat percentage were significantly reduced in the remission group after multidisciplinary management (*P* < 0.05). In comparison to those in the non-remission group, declines of BW and FBG were significantly pronounced in the remission group (*P* < 0.05).

**Table 3:** Changes in glucose metabolism, blood lipids, and body composition before and after 24 weeks of multidisciplinary management in the Diabetes Reversal Clinic (n=171).

Parameters	Before	After	*P*
Vital signs			
SBP (mmHg)	128.60 ± 15.33	125.62 ± 13.60	0.206
DBP (mmHg)	76.99 ± 10.64	75.86 ± 9.60	0.264
HR (bpm)	81.79 ± 11.37	81.41 ± 9.95	0.316
Glucose metabolism			
FBG (mmol/L)	7.45 (6.50, 9.00)	6.30 (5.63, 6.89)	<0.001
HbA1c (%)	7.30 (6.60, 8.70)	6.10 (5.78, 6.61)	<0.001
FINS (μU/mL)	8.97 (5.77, 12.34)	7.51 (4.70, 12.28)	0.227
HOMA-β	52.81 ± 41.24	64.67 ± 48.30	0.147
HOMA-IR	3.72 ± 2.67	2.67 ± 2.23	<0.001
FC-P (pmol/L)	780.52 ± 359.35	712.81 ± 309.59	0.732
Blood lipids			
TC (mmol/L)	4.75 ± 1.12	4.71 ± 1.07	0.687
TG (mmol/L)	1.48 (0.93, 2.29)	1.15 (0.83, 1.86)	0.387
HDL-C (mmol/L)	1.19 ± 0.32	1.31 ± 0.36	0.001
LDL-C (mmol/L)	2.97 ± 0.87	2.90 ± 0.86	0.867
Body composition			
BW (kg)	69.91 ± 12.45	67.55 ± 11.35	0.195
WC (cm)	88.05 ± 10.36	85.05 ± 8.33	0.003
BMI (kg/m^2^)	24.79 ± 3.27	23.98 ± 2.91	0.016
WHtR	0.53 ± 0.06	0.51 ± 0.05	<0.001
Body fat (kg)	19.18 ± 5.90	18.15 ± 7.07	0.516
Body fat percentage (%)	27.82 ± 7.54	25.24 ± 8.41	0.819
Skeletal muscle content (kg)	27.20 ± 5.40	29.93 ± 6.74	0.402
WHR	0.89 ± 0.05	0.89 ± 0.02	0.210
VFA (cm^2^)	94.19 ± 37.09	89.82 ± 47.97	0.502

T2DM, type 2 diabetes mellitus; SBP, systolic blood pressure; DBP, diastolic blood pressure; HR, heart rate; bpm, beats per minute; FBG, fasting blood glucose; HbA1c, glycated hemoglobin; FINS, fasting insulin; HOMA-β, homeostasis model assessment-beta cell function; HOMA-IR, homeostasis model assessment-insulin resistance; FC-P, fasting C-peptide; TC, total cholesterol; TG, triglyceride; HDL-C, high-density lipoprotein cholesterol; LDL-C, low-density lipoprotein cholesterol; BW, body weight; WC, waist circumference; BMI, body mass index; WHtR, waist-to-height ratio; WHR, waist hip rate; VFA, visceral fat area.

**Table 4:** Changes in glucose metabolism, blood lipids, and body composition before and after 24 weeks of multidisciplinary management of T2DM between the non-remission group and the remission group.

Parameters	Non-remission (n=121)			Remission (n=50)		
	Before	After	Change	Before	After	Change
Vital signs						
SBP (mmHg)	129.15 ± 16.62	126.47 ± 14.28	-2.68 ± 14.42	127.12 ± 11.25	123.31 ± 11.56	-4.16 ± 14.29
DBP (mmHg)	77.10 ± 10.57	75.74 ± 9.67	-1.36 ± 9.34	76.86 ± 10.71	76.24 ± 9.41	-1.12 ± 10.32
HR (bpm)	81.76 ± 11.27	82.84 ± 10.29	0.84 ± 12.94	81.67 ± 12.02	78.53 ± 8.75	-3.98 ± 12.38^#^
Glucose metabolism						
FBG (mmol/L)	8.58 ± 2.53	6.76 ± 1.77^***^	-1.97 ± 2.89	7.11 ± 1.98	5.96 ± 0.74^**^	-1.02 ± 2.09^##^
HbA1c (%)	7.95 ± 1.59	6.49 ± 0.87^***^	-1.46 ± 1.70	7.40 ± 2.07	5.82 ± 0.42^***^	-1.61 ± 2.29
FINS (μU/mL)	9.25 ± 5.06	8.91 ± 6.40	-0.99 ± 4.90	11.54 ± 7.69	9.93 ± 7.18	-2.41 ± 7.02
HOMA-β	38.09 (23.83, 54.72)	47.44 (28.38, 75.03)^*^	5.91 (-17.70, 23.27)	54.18 (39.32, 90.25)	54.12 (39.05, 90.25)	1.22 (-15.01, 33.33)
HOMA-IR	3.14 (2.19, 4.17)	2.13 (1.13, 3.40)^*^	-0.71 (-1.91, 0.15)	3.23 (1.91, 5.41)	1.72 (1.14, 3.40)^*^	-0.80 (-2.17, -0.01)
FC-P (pmol/L)	744.13 ± 312.86	690.88 ± 306.48	-60.00 ± 261.60	842.11 ± 424.03	763.06 ± 315.84	-101.80 ± 369.40
Blood lipids						
TC (mmol/L)	4.78 ± 1.08	4.77 ± 1.02	-0.04 ± 1.02	4.67 ± 1.25	4.57 ± 1.21	-0.05 ± 0.57
TG (mmol/L)	1.54 (1.09, 2.39)	1.27 (0.94, 2.02)^*^	-0.17 (-0.66, 0.09)	1.08 (0.68, 2.08)	0.94 (0.75, 1.36)	-0.11 (-0.43, 0.14)
HDL-C (mmol/L)	1.16 (1.00, 1.39)	1.23 (1.05, 1.50)^*^	0.09 (0.00, 0.20)	1.07 (0.95, 1.31)	1.29 (1.08, 1.47)^**^	0.17 (-0.04, 0.32)
LDL-C (mmol/L)	2.97 ± 0.83	2.91 ± 0.83	-1.11 ± 0.99	2.94 ± 0.96	2.88 ± 0.95	-0.05 ± 0.49
Body composition						
BW (kg)	69.13 ± 11.65	67.42 ± 11.03	-1.71 ± 3.81	71.81 ± 14.14	67.85 ± 12.22^***^	-3.66 ± 5.29^#^
WC (cm)	89.01 ± 8.74	85.91 ± 8.13^**^	-3.02 ± 4.39	85.40 ± 13.28	82.79 ± 8.38^***^	-2.47 ± 11.39
BMI (kg/m^2^)	24.63 ± 3.05	24.03 ± 2.83	-0.60 ± 1.38	25.17 ± 3.76	23.86 ± 3.13	-1.27 ± 1.85
WHtR	0.53 ± 0.05	0.51 ± 0.04^**^	-0.02 ± 0.03	0.49 ± 0.05	0.49 ± 0.05^*^	-0.01 ± 0.07
Body fat (kg)	18.40 ± 5.08	15.94 ± 5.59	-2.15 ± 2.02	20.61 ± 8.83	16.98 ± 7.20	-6.10 ± 5.51
Body fat percentage (%)	27.67 ± 6.89	23.81 ± 8.59	-2.46 ± 2.47	28.01 ± 9.01	22.65 ± 5.32^*^	-6.23 ± 6.57
Skeletal muscle content (kg)	26.52 ± 5.61	28.55 ± 6.34	-0.21 ± 1.19	28.88 ± 6.23	31.40 ± 6.44	1.48 ± 2.67
WHR	0.88 ± 0.04	0.87 ± 0.04	-0.01 ± 0.01	0.89 ± 0.05	0.87 ± 0.05	-0.01 ± 0.02
VFA (cm^2^)	97.27 ± 41.21	86.77 ± 34.98	-15.70 ± 17.52	96.74 ± 34.66	82.34 ± 56.73	-9.46 ± 56.46

^*^P < 0.05, ^**^P < 0.01, and ^***^P < 0.001, before vs. after multidisciplinary management; ^#^*P* < 0.05, ^##^*P* < 0.01, and ^###^*P* < 0.001, remission group vs. non-remission group.

T2DM, type 2 diabetes mellitus; SBP, systolic blood pressure; DBP, diastolic blood pressure; HR, heart rate; bpm, beats per minute; FBG, fasting blood glucose; HbA1c, glycated hemoglobin; FINS, fasting insulin; HOMA-β, homeostasis model assessment-beta cell function; HOMA-IR, homeostasis model assessment-insulin resistance; FC-P, fasting C-peptide; TC, total cholesterol; TG, triglyceride; HDL-C, high-density lipoprotein cholesterol; LDL-C, low-density lipoprotein cholesterol; BW, body weight; WC, waist circumference; BMI, body mass index; WHtR, waist-to-height ratio; WHR, waist hip rate; VFA, visceral fat area.

### Predictive factors for T2DM remission

**Figure 1:**
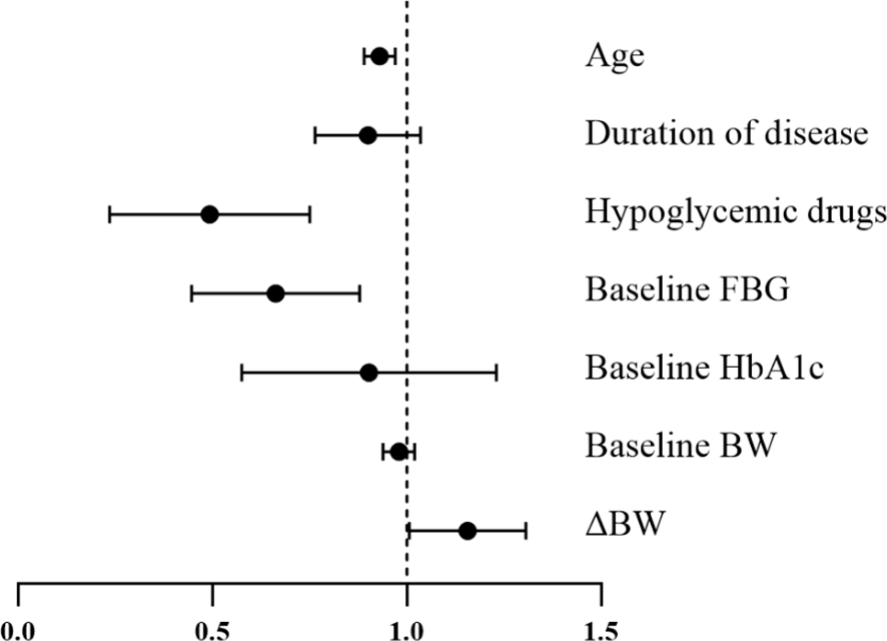
A forest map visualizing predictive factors for T2DM remission.

The multivariate logistic regression model showed that age (OR = 0.93, 95% CI: 0.89-0.97, *P* = 0.002), types of baseline hypoglycemic drugs (OR = 0.45, 95% CI: 0.26-0.77, *P* = 0.004), baseline FBG (OR = 0.64, 95% CI: 0.46-0.89, *P* = 0.008), and weight loss (OR = 1.15, 95% CI: 1.01-1.31, *P* = 0.038) were independent predictive factors for diabetes remission (**Table 5**). Their contributions to T2DM remission are visualized in **Figure 1**. It suggested that T2DM patients with a younger age, use of fewer baseline hypoglycemic drugs, lower baseline FBG, and greater weight loss after interventions were more likely to achieve remission.

**Table 5:** Multivariate logistic regression analysis of predictive factors for T2DM remission.

Influencing factors	OR	SE	95% CI	*P* value
Age (years)	0.93	0.02	0.89, 0.97	0.002
Duration of disease (years)	0.89	0.08	0.77, 1.04	0.141
Hypoglycemic drugs (n)	0.45	0.28	0.26, 0.77	0.004
Baseline FBG (mmol/L)	0.64	0.17	0.46, 0.89	0.008
Baseline HbA1c (%)	0.86	0.19	0.60, 1.25	0.438
Baseline BW (kg)	0.98	0.02	0.94, 1.02	0.276
ΔBW (kg)	1.15	0.07	1.01, 1.31	0.038

Δ represents the change before and after multidisciplinary management.

T2DM, type 2 diabetes mellitus; OR, odds ratio; SE, standard error; CI, confidence interval; HOMA-β, homeostasis model assessment-beta cell function; TG, triglyceride; FBG, fasting blood glucose; HbA1c, glycated hemoglobin; BW, body weight.

**Figure 2:**
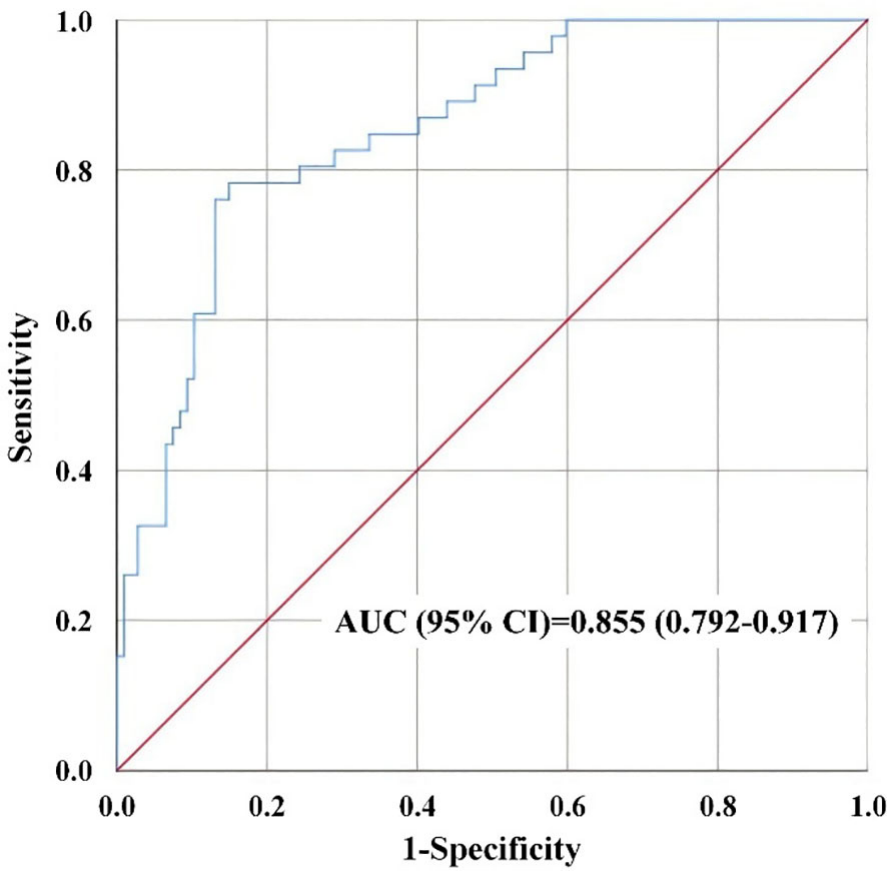
ROC curve of the multivariate model for T2DM remission.

**Figure 3:**
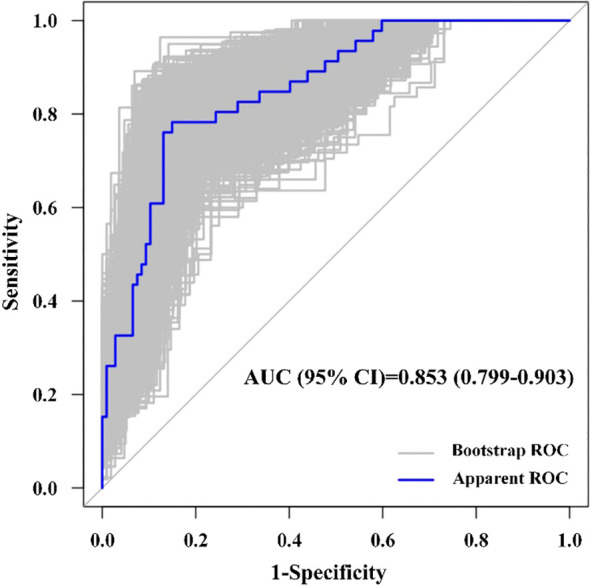
ROC curve after internal validation using Bootstrap resampling.

The model demonstrated good discrimination with an AUC of 0.855 (95% CI: 0.792-0.917, *P* < 0.001) in **Figure 2**. Based on the maximization of the Youden index (0.633), the optimal cut-off value for the model was determined to be 0.39, which yielded a sensitivity of 78.3% and a specificity of 85.0%. The Hosmer-Lemeshow goodness-of-fit test yielded a non-significant *P*-value of 0.354, indicating that the model fits the data well and there is no significant difference between the observed and predicted probabilities. Given the limited sample size, internal validation using Bootstrap resampling (with 1000 replicates) was conducted to evaluate model overfitting. The corrected AUC was calculated to be 0.853 (95% CI: 0.799-0.903), with the mean optimism of 0.002, indicating that the model possesses good stability (**Figure 3**).

### Safety outcomes

During the follow-up period, five hypoglycemic events were observed, three of which occurred in the basal insulin+GLP-1RA subgroup. Two patients experienced significant gastrointestinal discomfort, including diarrhea and nausea, both occurring during the initial phase of GLP-1RA treatment. No other notable adverse effects were observed. Furthermore, no significant safety issues were found during the period of medication withdrawal.

## Discussion

From April 12, 2022 to December 31, 2024, 171 eligible T2DM patients were regularly treated with remission-oriented multidisciplinary management in the Diabetes Reversal Clinic for 24 weeks and longer. The overall T2DM remission rate was 29.24% (50/171). Specifically, the highest remission rate was seen in T2DM patients with an oral intake of hypoglycemic drugs (37.65%), followed by those treated with GLP-1RA (35.29%), basal insulin (17.07%), and basal insulin+GLP-1RA (9.09%). Furthermore, T2DM patients presenting remission were significantly younger, showing a shorter duration of disease history, a use of fewer baseline hypoglycemic drugs, lower baseline FBG, HbA1c and TG, and a higher HOMA-β. Multivariate logistic regression identified that age, type of baseline hypoglycemic drugs, baseline FBG, and weight loss were factors independently associated with T2DM remission.

The observed overall remission rate of 29.24% in our study was higher than the rates reported in routine clinical care settings. Karter et al. reported a partial remission rate of 1.50%, a complete remission rate of 0.14% and a long-term remission rate of as low as 0.01% among 122,781 T2DM outpatients and inpatients receiving no bariatric surgery (21). In 2019, a cross-sectional study in Scotland found that the diabetes remission rate is only 4.8% among 162,316 T2DM patients registered in the health system (22). A latest study in Japan showed that among the 483,202 diabetes patients registered in the nationwide patient registry, the remission rate is 10.5 per 1000 person-years (23). Statistics from the English National Health Service (NHS) Type 2 Diabetes Path to Remission Programme reported a high diabetes remission rate of 32% (24), which similar with our data, emphasizing the importance of a specialized remission-targeted management of T2DM.

The T2DM remission rate was 37.65% in the oral hypoglycemic drug group, which is much higher than the 1-year remission rate of 26.7% reported previously in the same population (12). Li et al. reported a remission rate of 44% after 12-month interventions with 10 mg/d dapagliflozin plus calorie restriction in overweight/obese T2DM patients (25). All these data prove the efficacy of oral hypoglycemic drugs, although in different regimens, in inducing remission of T2DM. The exact role of GLP-1RA in T2DM remission has not been fully elucidated. A multi-center study in Canada showed that insulin glargine/lixisenatide combined with metformin and lifestyle interventions results in a 36-week remission rate of 31.6% among individuals within 5 years of T2DM diagnosis (26). In the present study, the diabetes remission rate was 35.29% in the GLP-1RA group, suggesting that GLP-1RA may favor glycemic control and remission. Mclnnes et al. demonstrated that basal insulin therapy yields a remission rate of 21.4% among T2DM patients diagnosed within 3 years (27), which is higher than the statistics (17.07%) we reported. The difference could be attributed to the duration of disease, while no limitation was set in our study. The lowest diabetes remission rate of 9.09% was observed in the basal insulin+GLP-1RA group, which had poor glycemic control at baseline (baseline FBG 10.13 ± 3.44 mmol/L, baseline HbA1c 8.95% ± 2.33%). Due to the small sample size (n=11), statistical comparisons involving this subgroup lacked sufficient power, and these findings should be interpreted with caution.

Changes in metabolic indicators before and after 24 weeks of multidisciplinary management were compared between the remission and non-remission groups. Indicators of glucose metabolism and blood lipids showed favorable changes in both groups, especially those related to glycemic control and insulin resistance. These improvements could be associated with the multidisciplinary management (e.g., individualized treatment regimen, diet and exercise interventions, health education, regular follow-up) in the Diabetes Reversal Clinic in T2DM remission. The changes in HOMA-β and TG were significant in the non-remission group, rather than the remission group, which could be explained by a better control of baseline blood glucose and lipids in the latter group. BW and body fat percentage were significantly reduced in the remission group, indicating that weight loss helps to achieve T2DM remission. A meta-analysis unveiled that the probability of complete diabetes remission increases by 2.17% for every 1% weight loss (28).

We further compared clinical characteristics between the remission group and non-remission group. Compared with the non-remission group, the T2DM patients in the remission group were significantly younger, with a shorter disease duration, a use of fewer hypoglycemic drugs, lower baseline FBG, HbA1c, and TG, a higher baseline HOMA-β, and greater declines of FBG and BW after interventions. These univariate comparisons suggest that factors such as the younger age, shorter duration of disease, use of fewer baseline hypoglycemic drugs, lower blood sugar and lipids, better islet function, and greater FBG decline and weight loss could be associated with T2DM remission. However, after adjusting for potential confounders in the multivariate logistic regression, only age, number of baseline hypoglycemic drugs, baseline FBG, and weight loss remained independently associated with remission, disease duration did not retain statistical significance. The DiRECT study consistently unveiled a close correlation of age, duration of disease, HbA1c and islet function with diabetes remission (29). The lack of independent association for disease duration and HbA1c in our model may be attributed to the limited sample size.

This study has several limitations that warrant consideration. First, as an observational study with a single-arm design and no control group, we cannot definitively establish a causal relationship between the multidisciplinary management and the observed remission rate. Second, there is a potential for selection bias, as patients who chose to attend the Diabetes Reversal Clinic are likely highly motivated to achieve remission, which may not be representative of the general T2DM population and could lead to an overestimation of the remission rate. Third, the relatively small sample size (n=171) limits the statistical power for subgroup analyses and the detection of small but clinically meaningful effects, and may have influenced the results of the multivariate regression model. Fourth, the follow-up period of 24 weeks is relatively short, capturing only short-term remission. The long-term sustainability of remission and the rate of relapse require further investigation with extended follow-up (≥1–2 years). Finally, despite adjusting for potential confounders in the regression model, there may be unmeasured confounding factors that could have influenced the results.

Looking forward, an integration of artificial intelligence (AI) and digital tools (e.g., online diabetes management apps) with multidisciplinary management in the Diabetes Reversal Clinic is expected to elevate the remission rate. AI, as a cutting-edge technique, can assist in the real-time modification of individualized treatment regimens, and the prediction of diabetes remission, thereafter optimizing medical resource allocation (30). Online apps can provide easy accesses to record physiological parameters wherever and whenever possible. They also allow remote guidance of diet and exercise, and timely adjustment of treatment regimens (31). These attempts, in combination with multidisciplinary management in the Diabetes Reversal Clinic, may enhance patient compliance, increase long-term T2DM remission rate, as well as reduce the risk of diabetes-related complications. However, the efficacy and cost-effectiveness of such digital augmentations require rigorous evaluation in prospective studies.

In conclusion, the multidisciplinary management for more than 24 weeks in the Diabetes Reversal Clinic was associated with a T2DM remission rate of 29.24%. Age, type of baseline hypoglycemic drugs, baseline FBG, and weight loss were identified as independent factors associated with remission. Our experience provides a potentially effective model for achieving diabetes remission in outpatient clinics of China. However, it is important to acknowledge the limitations of this study, including its observational design, lack of a control group, relatively small sample size, and short follow-up period, which prevent definitive causal inferences about the effectiveness of the intervention. Therefore, larger, prospective studies with longer follow-up periods are needed to validate these findings, confirm the long-term sustainability of remission, and further elucidate the optimal components of multidisciplinary management for T2DM remission.

## Data Availability

The raw data supporting the conclusions of this article will be made available by the authors, without undue reservation.
